# Do individual and work-related factors differentiate work participation trajectories before and after vocational rehabilitation?

**DOI:** 10.1371/journal.pone.0212498

**Published:** 2019-02-21

**Authors:** Taina Leinonen, Svetlana Solovieva, Kirsti Husgafvel-Pursiainen, Mikko Laaksonen, Eira Viikari-Juntura

**Affiliations:** 1 Finnish Institute of Occupational Health, Helsinki, Finland; 2 Finnish Centre for Pensions, Helsinki, Finland; University of Melbourne, AUSTRALIA

## Abstract

**Background:**

Understanding diverse labor market trajectories around vocational rehabilitation provides important insight into potential effectiveness of rehabilitation efforts. We examined factors associated with work participation trajectories before and after vocational rehabilitation.

**Methods:**

Using nationwide Finnish register data of 7180 vocational rehabilitees, we constructed latent trajectory groups of work participation two years before and two years after their rehabilitation episode starting in 2008–2010. We plotted changes in labor market statuses in these groups and examined other associated factors using multinomial logistic regression.

**Results:**

We identified four trajectories based on work participation levels before and after vocational rehabilitation. The “High–Resumed” group (35.6%) typically returned to full duties. The “High–to–Negligible” group (20.7%) typically transitioned to full disability retirement or unemployment. Among the “Medium–Resumed” (25.5%) and “Longstanding Negligible” (18.3%) groups, work disability and unemployment were common before rehabilitation, but afterwards those assigned to the former group often returned to full or partial duties. Overall, older age, male gender, living in areas with lower employment rates, having lower education, being employed in the private sector, and having mental diagnoses were associated with the other three trajectories than the most favorable “High-Resumed” trajectory. Furthermore, certain industrial sectors, job exposures, and less common diagnoses further separated specific trajectories.

**Conclusions:**

Work participation trajectories around vocational rehabilitation are diverse, only partly dependent on initial levels of work participation, and determined by various individual and work-related factors. Future nationwide studies should assess the effectiveness of vocational rehabilitation taking into consideration both individual and work-related factors.

## Introduction

Labor market outcomes after vocational rehabilitation may depend on various characteristics of the rehabilitee such as labor market history, the nature of work disability, and sociodemographic factors. More favorable employment outcomes have generally been reported for individuals who are younger, have a higher socioeconomic position, have more previous employment, and have shorter length of preceding disability, while variation by other factors such as gender and disease groups remains unclear [1–10].

Assessment of labor market outcomes after vocational rehabilitation is complex, because return to work may be perceived as a multiphase and multifaceted process rather than a single event [11–13]. There is a large number of possible transitions between different labor market statuses around rehabilitation [14–17] as well as potential variation between shorter- and longer-term outcomes [10,18,19]. Moreover, the close link between a rehabilitee’s preceding labor market history and subsequent labor market outcomes may lead to biased conclusions of the effects of vocational rehabilitation.

Investigating labor market trajectories over a lengthy period of time surrounding vocational rehabilitation provides important insight into the factors associated with potential effectiveness of rehabilitation efforts. While previous studies have examined changes between different labor market statuses after vocational or other work-related rehabilitation [14–18], less is known of labor market trajectories covering the period both before and after vocational rehabilitation [19]. Furthermore, predictors of such trajectories remain unclear.

We used nationally representative Finnish register data to identify latent trajectory groups of work participation over a period of two years before and two years after an episode of vocational rehabilitation. In order to find out which statuses the individuals in question occupied while being out of work, we further examined changes in the receipt of different social security benefits among the different trajectory groups. In addition, we examined factors associated with being assigned to particular trajectory group of work participation around vocational rehabilitation. More specifically, we aimed to answer the following research questions:

1. What kind of typical work participation patterns can be found around vocational rehabilitation?
2. What kind of changes can be observed in statuses other than work, such as sickness absence, disability retirement, and unemployment, around vocational rehabilitation among groups following different work participation trajectories?
3. Are sociodemographic factors, prior work-related exposures, and characteristics of the rehabilitation episode associated with particular work participation trajectories around vocational rehabilitation?

## Material and methods

### Study design

We used a nationally representative 70% random sample of the working aged population living in Finland on the last day of year 2007. The register-based data include information on episodes of vocational rehabilitation, employment, unemployment, earnings-related retirement, and other benefit receipt from the Finnish Centre for Pensions, on episodes of compensated sickness absence and national pensions obtained from the Finnish Social Insurance Institution, and on sociodemographic and work-related factors obtained from the Finnish Longitudinal Employer‒Employee Data (FLEED). For the purposes of this study, we utilized information from calendar years 2006–2014.

Since 2004, vocational rehabilitation has been a statutory right in Finland. Eligibility to vocational rehabilitation is based on evaluation of a threat of disability retirement within the next few years due to a diagnosed illness or an injury as well as of the expectation that work participation can be promoted and disability retirement postponed or prevented with vocational rehabilitation. The system emphasizes early onset of rehabilitation in order to reach these goals. Vocational rehabilitation in Finland is highly fragmented. Those who are attached to working life with a sufficient amount of recent employment (sum of earnings in the previous five years approximately 35000 euros at 2017 level) are eligible for vocational rehabilitation provided by the earnings-related pension scheme, while those who are outside working life may receive vocational rehabilitation from the Social Insurance Institution of Finland or other sources [20–22].

We studied only vocational rehabilitation that was provided by the earnings-related pension scheme, the rehabilitees therefore being relatively well attached to the labor market. This does not mean that all rehabilitees came directly from employment. In 2016, 23% came to vocational rehabilitation from retirement, typically after having received a temporarily granted disability pension [23]. The main types of vocational rehabilitation provided by the earnings-related pension scheme include training, work counselling, and work try-outs. Work try-outs are the most prevalent type and often carried out at a person’s own workplace. Medical rehabilitation is not covered in the earnings-related pension scheme [21]. Our data did not include information on medical rehabilitation or treatment.

We included individuals whose vocational rehabilitation episode began in 2008–2010 and who were aged 25–59 at that time. Daily changes in work participation and other labor market statuses were followed up over a period of two years before the begin date and two years after the end date of vocational rehabilitation. This four-year measurement period of labor market participation did not cover the time period in the middle that was spent in vocational rehabilitation. However, because our data on labor market participation was available until the end of October 2014, and because we wanted to follow-up each study person over a full two-year period after the termination of their rehabilitation episode, we could only examine rehabilitation episodes that ended by October 2012, i.e. 22 months after the last episodes of year 2010 had begun. We therefore excluded individuals whose vocational rehabilitation episode lasted more than 22 months (24.1%). The final study population consisted of 7180 individuals.

### Work participation and other labor market statuses

Work participation trajectories was the outcome of main interest. We used information on episodes of employment and of receiving social security benefits to calculate the monthly proportion of time spent at work over the 24 months before and 24 months after vocational rehabilitation. We assumed that those who had an ongoing employment episode without receiving any benefits that compensated for being out of work, such as sickness allowance, worked 100% of the time; full-time work is very typical in Finland [24]. We also assumed that individuals receiving a partial sickness allowance or a partial disability pension (together referred to as partial work disability benefits) worked 50% of the time. In Finland, receipt of the partial sickness allowance requires the person to work 40–60% of the time.

We further examined changes in six mutually exclusive labor market statuses over the two years before and two years after vocational rehabilitation. By doing so, we could assess how overall work participation was divided into full and partial duties as well as what were the prevalent statuses while being fully out of work. The statuses were 1) work (with full work duties), 2) partial work disability (with partial work duties), 3) full sickness absence, 4) unemployment, 5) full disability retirement (temporary or permanent), and 6) other (e.g. education, parental leave, old-age retirement, or dead).

### Covariates

We included sociodemographic factors (age, gender, region of residence, and education), prior work-related exposures (industrial sector and various job exposures based on previously developed job exposure matrices), and characteristics of the vocational rehabilitation episode (employment sector, disease group, start year, and duration) as covariates. Age at the start of rehabilitation was examined in groups 25–39, 40–49, and 50–59 years. Region of residence and education were measured at the end of the year preceding the measurement period of labor market participation. Region of residence consisted of categories 1) Southern, 2) Western (including the Åland islands), 3) Eastern, and 4) Northern Finland. Education consisted of categories 1) tertiary, 2) secondary, and 3) primary.

Industrial sector and occupational information for the job exposure matrices were also primarily measured at the end of the year preceding the measurement period of labor market participation. For those who were non-employed in that year, however, the information was derived from the end of other years, available for industrial sector over a five-year period and for occupation over a four-year period preceding the start year of vocational rehabilitation. Priority was nevertheless given to deriving the information from the years preceding the measurement period of labor market participation. Industrial sector and occupation could not be identified for 160 and 285 individuals, respectively, because they were constantly non-employed before rehabilitation. These individuals were excluded from the models including industrial sector or job exposures.

Industrial sector was based on a classification by Statistics Finland. We examined the following categories as dummy variables: 1) manufacturing, 2) trade (wholesale and retail trade; repair of motor vehicles and motorcycles), 3) transportation and storage, 4) knowledge work (information and communication; financial and insurance activities; real estate activities; professional, scientific and technical activities), and 5) human health and social work activities.

For occupation, we used a classification by Statistics Finland, based on the International Standard Classification of Occupations (ISCO-88). Job exposures were then estimated for each occupation with a gender-specific job exposure matrix (JEM), which was developed earlier in a large population survey and described with more detail elsewhere [25,26]. Heavy physical work, kneeling and squatting, as well as repetitive hand movements could receive values between 0 and 100 (highest). For heavy physical work as well as kneeling and squatting, the frequencies of exposure peaked at 0 and at a little above 40. We classified these exposures as 1) none (0), 2) low (>0, <40), and 3) high (≥40). Repetitive hand movements had a more even distribution and was used as a continuous variable. Job strain was based on the Karasek model [27] including the categories 1) low strain (low job demands, high job control), 2) active job (high job demands, high job control), 3) passive job (low job demands, low job control), and 4) high strain (high job demands, low job control). Monotonous work included the categories 1) no and 2) yes.

Employment sector that provided the vocational rehabilitation was classified as 1) private and 2) public. In the national vocational rehabilitation scheme, the provider is selected based on current or last employment.

The primary medical reason for vocational rehabilitation was classified according to the tenth revision of the International Classification of Diseases (ICD-10). We examined the following disease groups as dummy variables: 1) musculoskeletal diseases (M00–M99), 2) mental disorders (F00–F99), 3) neoplasms (C00–D48), 4) nervous diseases (G00–G99), 5) circulatory diseases (I00–I99), and 6) injuries (injury, poisoning and certain other consequences of external causes, S00–T98).

Duration of vocational rehabilitation was based on the total continuous duration and could consist of various successive vocational rehabilitation efforts of different types. A large peak was observed at the duration corresponding to around three months, and smaller peaks at the duration corresponding to four, five, and six months. We categorized duration into 1) <3 months, 2) exactly 3 months (equaling 89–93 days), 3) >3 months, ≤6 months, and 4) >6 months.

### Statistical analyses

We constructed work participation trajectories based on monthly measured work participation over two years before and two years after the episode of vocational rehabilitation. The analysis time was assessed as a single four-year period, excluding the time spent in rehabilitation. The work participation trajectories were obtained using a semiparametric group-based modelling strategy by PROC TRAJ in SAS version 9.4 (SAS Institute, Cary, NC, USA). This method was developed for analyzing longitudinal data, changes over time, and identifying distinct latent groups of subjects who tend to have a similar profile over time (trajectories) [28,29]. The Bayesian information criterion (BIC) was considered when selecting the optimal model, number of trajectories and their shape. With continuous data, the normal distribution was used as the underlying statistical model.

After constructing the work participation trajectories, we plotted changes in the distribution of the six labor market statuses before and after vocational rehabilitation among the different trajectory groups of work participation.

We then examined how the different covariates were associated with assignment to different trajectory groups of work participation around vocational rehabilitation using multinomial logistic regression analysis. We calculated relative risk ratios (RRR) and their 95% confidence intervals.

### Ethics statement

The study was fully register-based and applied identification numbers pseudonymized by Statistics Finland. Research using such data does not need to undergo review by an ethics committee according to Finnish legislation. The researchers analyzed the data stored by Statistics Finland using a remote access system. All output extracted from the system was approved by Statistics Finland to ensure compliance with data protection regulations.

## Results

Over 40% of the rehabilitees were aged 50 or above and over 60% were women. Close to half of the study population came to rehabilitation because of musculoskeletal diseases and over a fourth because of mental disorders. Further characteristics of the rehabilitees are presented in Table 1.

**Table 1:** Distribution of background characteristics among the vocational rehabilitees.

	N	%	Range	Mean
Age			25–59	46.2
25–39	1522	21.2		
40–49	2737	38.1		
50–59	2921	40.7		
Gender				
Women	4507	62.8		
Men	2673	37.2		
Region of residence				
South	3239	45.1		
West	1713	23.9		
East	1092	15.2		
North	1136	15.8		
Education				
Tertiary	1859	25.9		
Secondary	3910	54.5		
Primary	1411	19.7		
Industrial sector				
Manufacturing	1188	16.6		
Trade	644	9.0		
Transportation & storage	623	8.7		
Knowledge work	597	8.3		
Health & social work	1627	22.7		
Other	2341	32.6		
No industrial sector identified	160	2.2		
Heavy physical work			0–100	37.1
None	517	7.2		
Low	2906	40.5		
High	3472	48.4		
No occupation identified	285	4.0		
Kneeling and squatting at work			0–96.7	26.9
None	805	11.2		
Low	3821	53.2		
High	2269	31.6		
No occupation identified	285	4.0		
Repetitive hand movements at work			0–100	45.3
Occupation identified for calculation	6895	96.0		
No occupation identified	285	4.0		
Job strain				
Low strain	1119	15.6		
Active job	709	9.9		
Passive job	3289	45.8		
High strain	1778	24.8		
No occupation identified	285	4.0		
Monotonous work				
No	4658	64.9		
Yes	2237	31.2		
No occupation identified	285	4.0		
Sector of rehabilitation				
Private	4640	64.6		
Public	2540	35.4		
Disease group of rehabilitation				
Musculoskeletal	3393	47.3		
Mental	1942	27.1		
Neoplasms	219	3.1		
Nervous	350	4.9		
Circulatory	290	4.0		
Injuries	464	6.5		
Other	522	7.3		
Start year of rehabilitation				
2008	2333	32.5		
2009	2352	32.8		
2010	2495	34.8		
Duration of rehabilitation			0.1–22.0	5.2
<3 months	1474	20.5		
3 months	2392	33.3		
>3 months, ≤6 months	1826	25.4		
>6 months	1488	20.7		
Total	7180	100.0		

### Construction of the trajectory groups of work participation

**Fig 1:**
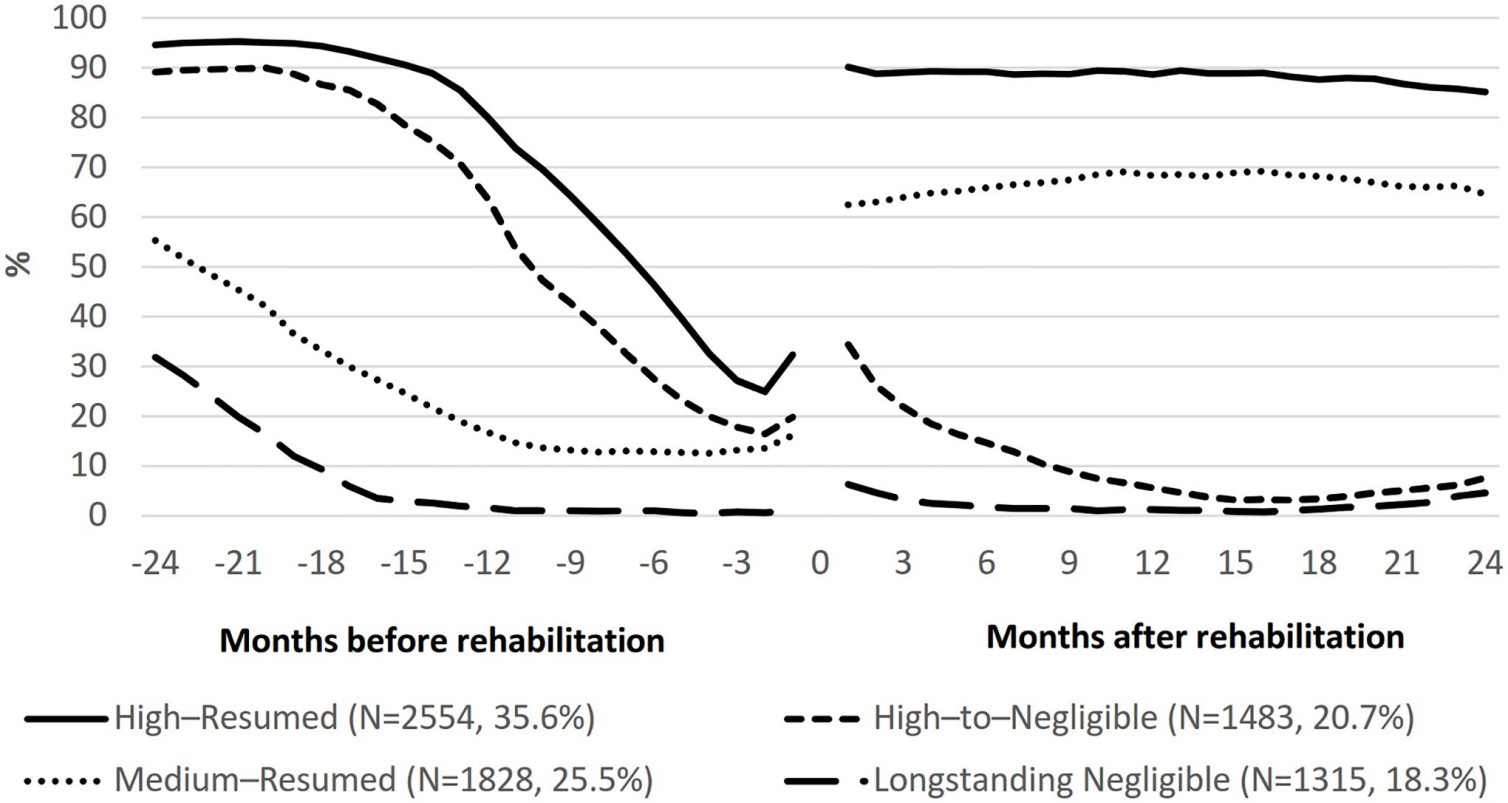
Four identified trajectory groups of work participation over the period of two years before and two years after vocational rehabilitation.

Four trajectory groups of work participation around vocational rehabilitation were identified (Fig 1). In the first group (35.6%), work participation was initially at a high level, declined particularly during a one-year period before vocational rehabilitation, and resumed close to its initial level immediately after vocational rehabilitation (High–Resumed group). In the second group (20.7%), work participation was initially at a high level, declined during a 1.5-year period before vocational rehabilitation, increased only slightly and momentarily after vocational rehabilitation, and then again declined reaching a negligible level by the end of the first year after vocational rehabilitation (High–to–Negligible group). In the third group (25.5%), work participation was initially at a medium level, declined particularly in the period 2–1 years before vocational rehabilitation, and resumed its initial level or even slightly above immediately after vocational rehabilitation (Medium–Resumed group). In the fourth group (18.3%), work participation was initially at a low level, declined reaching a negligible level already more than one year before vocational rehabilitation, increased only very slightly and momentarily after vocational rehabilitation, and then rapidly declined back to the negligible level (Longstanding Negligible group).

### Changes in labor market status among the trajectory groups

**Fig 2:**
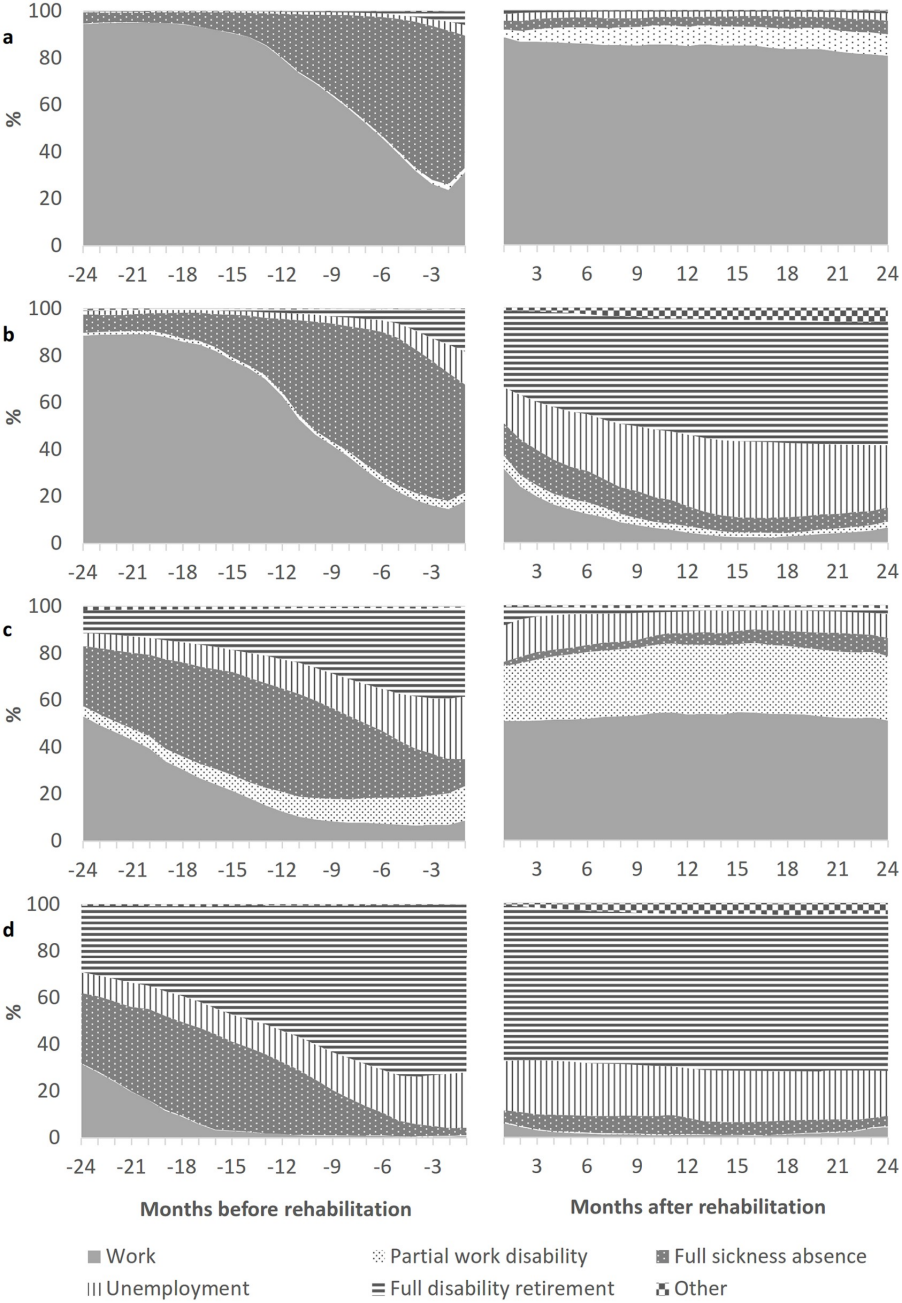
Changes in labour market status over the period of two years before and two years after vocational rehabilitation among the trajectory groups of work participation: a) High–Resumed (N = 2554), b) High–to–Negligible (N = 1483), c) Medium–Resumed (N = 1828), and d) Longstanding Negligible (N = 1315).

Among the High–Resumed trajectory group, the decrease in work participation before vocational rehabilitation was mainly replaced by a corresponding increase in full sickness absence (Fig 2a). The resumed high level of work participation after rehabilitation was mainly attributed to by return to full duties and to a relatively small extent by return to partial duties while receiving partial disability benefits.

The decrease in work participation before vocational rehabilitation among the High–to–Negligible group was also mainly replaced by an increase in full sickness absence, but also by small increases in unemployment and full disability retirement (Fig 2b). After vocational rehabilitation, full disability retirement was the most common status followed by unemployment. Partial work disability was not common among this group.

Among the Medium–Resumed group, full sickness absence was a common status particularly in the period 2–0.5 years before vocational rehabilitation (Fig 2c). Partial work disability somewhat increased before rehabilitation. Furthermore, the decrease in work participation before vocational rehabilitation was countered by increases in full disability retirement and unemployment. The resumed medium level of work participation after rehabilitation was attributed to not only by return to full duties, but to a relatively large extent also by return to partial duties while receiving partial disability benefits. Unemployment also remained relatively common after rehabilitation.

The Longstanding Negligible group was mainly on full disability retirement both before and after vocational rehabilitation (Fig 2d). Also sickness absence in the period 2–0.5 years before rehabilitation and unemployment both before and after rehabilitation were common statuses. Partial work disability was virtually nonexistent among this group.

### Predictors of assignment to the trajectory groups

First, we assessed age- and gender-adjusted predictors of being assigned to the High–to–Negligible, the Medium–Resumed, and the Longstanding Negligible trajectory groups of work participation around vocational rehabilitation, compared to being assigned to the most favorable group, i.e. the High–Resumed group (Table 2). Male gender, having less than tertiary education, and being rehabilitated due to mental disorders predicted assignment to all of the three less favorable trajectory groups. The influence of education was particularly strong with respect to the Longstanding Negligible trajectory. Furthermore, many common factors predicted assignment to the High–to–Negligible and Longstanding Negligible trajectory groups, including age 50–59, living in Eastern Finland, employment in the trade sector, repetitive hand movements at work, high job strain, being rehabilitated in the private rather than in the public sector, and generally also being rehabilitated during either shorter or longer periods than the common period of exactly three months. Some common factors also predicted assignment to both the Medium–Resumed and the Longstanding Negligible trajectory groups, such as living in Northern Finland, exposure to kneeling and squatting at work, and starting rehabilitation in year 2008. Other factors were associated with one of the trajectories specifically: employment in the manufacturing sector, monotonous work, and being rehabilitated due to nervous diseases with the High–to–Negligible trajectory, being rehabilitated due neoplasms and injuries with the Medium–Resumed trajectory, and exposure to heavy physical work with the Longstanding Negligible trajectory.

**Table 2:** Age- and gender-adjusted predictors of being assigned to different trajectory groups of work participation around vocational rehabilitation.

	High–to–Negligible		Medium–Resumed		Longstanding Negligible	
	vs.		vs.		vs.	
	High–Resumed		High–Resumed		High–Resumed	
	RRR	95% CI	RRR	95% CI	RRR	95% CI
Age^a^						
25–39	1.00		1.00		1.00	
40–49	1.10	(0.92–1.32)	1.18	(1.01–1.38)	1.07	(0.89–1.28)
50–59	2.05	(1.72–2.44)	1.12	(0.95–1.31)	1.45	(1.21–1.73)
Gender^a^						
Women	1.00		1.00		1.00	
Men	1.44	(1.26–1.65)	1.17	(1.03–1.32)	1.48	(1.29–1.70)
Region of residence^a^						
South	1.00		1.00		1.00	
West	0.97	(0.82–1.14)	1.09	(0.93–1.27)	1.18	(1.00–1.40)
East	1.33	(1.10–1.61)	1.17	(0.97–1.40)	1.39	(1.14–1.69)
North	1.16	(0.96–1.41)	1.48	(1.24–1.76)	1.58	(1.30–1.92)
Education^a^						
Tertiary	1.00		1.00		1.00	
Secondary	1.39	(1.19–1.62)	1.22	(1.06–1.41)	1.71	(1.44–2.02)
Primary	1.59	(1.30–1.93)	1.53	(1.28–1.84)	2.25	(1.83–2.77)
Industrial sector^b^ (dummies)						
Manufacturing	1.68	(1.39–2.03)	1.02	(0.84–1.24)	1.09	(0.88–1.34)
Trade	1.65	(1.30–2.09)	1.15	(0.91–1.46)	1.54	(1.20–1.98)
Transportation & storage	1.05	(0.82–1.34)	0.93	(0.74–1.17)	0.76	(0.58–0.99)
Knowledge work	1.01	(0.80–1.29)	0.78	(0.62–0.99)	0.77	(0.59–1.01)
Health & social work	0.83	(0.69–1.01)	1.18	(1.00–1.39)	1.03	(0.85–1.25)
Heavy physical work^c^						
None	1.00		1.00		1.00	
Low	1.11	(0.86–1.43)	1.19	(0.94–1.51)	1.73	(1.27–2.37)
High	1.24	(0.96–1.60)	1.24	(0.98–1.58)	1.89	(1.39–2.58)
Kneeling and squatting at work^c^						
None	1.00		1.00		1.00	
Low	1.10	(0.89–1.35)	1.27	(1.04–1.56)	1.31	(1.03–1.67)
High	1.09	(0.88–1.36)	1.45	(1.18–1.79)	1.70	(1.32–2.18)
Repetitive hand movements at work^c^ (for 10% increase)	1.05	(1.02–1.07)	1.00	(0.98–1.03)	1.05	(1.02–1.07)
Job strain^c^						
Low strain	1.00		1.00		1.00	
Active job	1.14	(0.88–1.48)	0.98	(0.78–1.24)	0.96	(0.71–1.28)
Passive job	1.17	(0.97–1.42)	0.96	(0.81–1.14)	1.22	(0.99–1.50)
High strain	1.53	(1.24–1.89)	1.18	(0.98–1.43)	1.56	(1.24–1.95)
Monotonous work^c^						
No	1.00		1.00		1.00	
Yes	1.34	(1.17–1.54)	1.14	(1.00–1.31)	1.17	(1.00–1.36)
Sector of rehabilitation^a^						
Private	1.00		1.00		1.00	
Public	0.62	(0.54–0.72)	0.90	(0.79–1.03)	0.72	(0.62–0.84)
Disease group of rehabilitation^a^ (dummies)						
Musculoskeletal	1.25	(0.96–1.61)	1.17	(0.92–1.49)	1.18	(0.90–1.55)
Mental	1.32	(1.01–1.74)	1.34	(1.04–1.72)	1.63	(1.23–2.15)
Neoplasms	1.24	(0.79–1.95)	1.69	(1.13–2.52)	1.27	(0.79–2.04)
Nervous	1.67	(1.16–2.42)	1.29	(0.90–1.83)	1.18	(0.79–1.78)
Circulatory	1.20	(0.82–1.77)	0.85	(0.58–1.25)	0.91	(0.59–1.39)
Injuries	0.93	(0.65–1.35)	1.42	(1.03–1.95)	1.32	(0.92–1.89)
Start year of rehabilitation^a^						
2008	1.00		1.00		1.00	
2009	0.95	(0.81–1.12)	0.90	(0.77–1.04)	0.79	(0.67–0.93)
2010	0.88	(0.75–1.03)	0.83	(0.72–0.96)	0.75	(0.64–0.89)
Duration of rehabilitation^a^						
<3 months	1.60	(1.33–1.91)	0.74	(0.62–0.88)	1.75	(1.46–2.10)
3 months	1.00		1.00		1.00	
>3 months, ≤6 months	1.37	(1.15–1.63)	1.04	(0.90–1.22)	1.03	(0.86–1.24)
>6 months	1.34	(1.11–1.61)	0.99	(0.83–1.17)	1.29	(1.07–1.56)

^a^ Including all: N = 7180.

^b^ Including those for whom industrial sector was identified: N = 7020.

^c^ Including those for whom occupation was identified: N = 6895.

We then assessed mutually adjusted predictors of being assigned to the different trajectory groups (Table 3). The analyses included only those for whom industrial sector and occupation could be identified. Most of the associations were similar to those in the age- and gender-adjusted models. However, employment in the health and social work sector now predicted assignment to the Medium–Resumed and the Longstanding Negligible trajectory groups. The associations between employment in the manufacturing and trade sectors with the High–to–Negligible trajectory nevertheless remained. With respect to job exposures, only the associations of high exposure to kneeling and squatting with the Medium–Resumed trajectory and of high job strain with the Longstanding Negligible trajectory remained. In addition, compared to the High–Resumed group, being rehabilitated in the private sector now predicted assignment to each one of the other three trajectory groups.

**Table 3:** Mutually adjusted predictors of being assigned to different trajectory groups of work participation around vocational rehabilitation.

	High–to–Negligible		Medium–Resumed		Longstanding Negligible	
	vs.		vs.		vs.	
	High–Resumed		High–Resumed		High–Resumed	
	RRR	95% CI	RRR	95% CI	RRR	95% CI
Age						
25–39	1.00		1.00		1.00	
40–49	1.17	(0.97–1.41)	1.22	(1.04–1.44)	1.19	(0.98–1.45)
50–59	2.25	(1.87–2.71)	1.19	(1.00–1.41)	1.76	(1.44–2.14)
Gender						
Women	1.00		1.00		1.00	
Men	1.21	(1.03–1.42)	1.18	(1.01–1.37)	1.27	(1.07–1.52)
Region of residence						
South	1.00		1.00		1.00	
West	0.96	(0.81–1.13)	1.08	(0.93–1.27)	1.13	(0.94–1.36)
East	1.36	(1.12–1.65)	1.15	(0.95–1.39)	1.30	(1.05–1.61)
North	1.17	(0.96–1.42)	1.49	(1.24–1.78)	1.46	(1.19–1.80)
Education						
Tertiary	1.00		1.00		1.00	
Secondary	1.30	(1.08–1.56)	1.24	(1.04–1.47)	1.65	(1.33–2.03)
Primary	1.43	(1.14–1.79)	1.60	(1.29–1.98)	2.25	(1.75–2.89)
Industrial sector (dummies)						
Manufacturing	1.53	(1.24–1.90)	0.94	(0.76–1.16)	0.94	(0.74–1.19)
Trade	1.59	(1.21–2.09)	1.01	(0.77–1.33)	1.25	(0.93–1.67)
Transportation & storage	0.92	(0.69–1.23)	0.80	(0.60–1.05)	0.67	(0.48–0.92)
Knowledge work	1.03	(0.79–1.34)	0.80	(0.62–1.03)	0.78	(0.58–1.05)
Health & social work	0.99	(0.79–1.25)	1.36	(1.11–1.66)	1.31	(1.03–1.67)
Heavy physical work						
None	1.00		1.00		1.00	
Low	1.08	(0.76–1.53)	0.88	(0.63–1.23)	1.36	(0.90–2.07)
High	1.14	(0.78–1.68)	0.80	(0.55–1.16)	1.19	(0.76–1.87)
Kneeling and squatting at work						
None	1.00		1.00		1.00	
Low	0.92	(0.69–1.24)	1.27	(0.95–1.69)	1.06	(0.76–1.48)
High	0.97	(0.70–1.33)	1.39	(1.02–1.90)	1.24	(0.87–1.76)
Repetitive hand movements at work (for 10% increase)	0.99	(0.96–1.03)	1.00	(0.96–1.03)	1.02	(0.98–1.06)
Job strain						
Low strain	1.00		1.00		1.00	
Active job	1.18	(0.90–1.56)	1.05	(0.81–1.35)	1.08	(0.79–1.48)
Passive job	0.92	(0.74–1.15)	0.91	(0.75–1.12)	1.06	(0.83–1.35)
High strain	1.18	(0.91–1.53)	1.15	(0.90–1.47)	1.35	(1.02–1.80)
Monotonous work						
No	1.00		1.00		1.00	
Yes	1.12	(0.92–1.36)	1.18	(0.98–1.43)	1.01	(0.81–1.24)
Sector of rehabilitation						
Private	1.00		1.00		1.00	
Public	0.78	(0.64–0.95)	0.84	(0.70–0.99)	0.68	(0.56–0.84)
Disease group of rehabilitation (dummies)						
Musculoskeletal	1.14	(0.87–1.48)	1.20	(0.93–1.54)	1.21	(0.90–1.63)
Mental	1.53	(1.15–2.03)	1.58	(1.22–2.07)	2.14	(1.56–2.92)
Neoplasms	1.34	(0.84–2.12)	1.91	(1.26–2.89)	1.58	(0.95–2.63)
Nervous	1.65	(1.13–2.41)	1.30	(0.90–1.88)	1.33	(0.86–2.06)
Circulatory	1.25	(0.85–1.86)	0.98	(0.66–1.46)	1.18	(0.75–1.86)
Injuries	0.89	(0.61–1.30)	1.48	(1.06–2.06)	1.31	(0.89–1.95)
Start year of rehabilitation						
2008	1.00		1.00		1.00	
2009	0.94	(0.80–1.11)	0.91	(0.78–1.06)	0.81	(0.68–0.96)
2010	0.90	(0.76–1.05)	0.83	(0.72–0.97)	0.77	(0.64–0.91)
Duration of rehabilitation						
<3 months	1.61	(1.34–1.93)	0.73	(0.61–0.88)	1.72	(1.42–2.08)
3 months	1.00		1.00		1.00	(1.42–2.08)
>3 months, ≤6 months	1.30	(1.09–1.55)	1.04	(0.89–1.22)	0.96	(0.78–1.17)
>6 months	1.26	(1.04–1.53)	1.00	(0.84–1.19)	1.28	(1.04–1.57)

Including those for whom industrial sector and occupation was identified: N = 6895.

## Discussion

### Main findings and their interpretations

Using a nationally representative sample of vocational rehabilitees, we identified four typical work participation trajectories over a period of two years before and two years after their episode of vocational rehabilitation that started in 2008–2010. Over one third and around one fourth of the rehabilitees followed trajectories where initial high or initial medium level of work participation, respectively, was resumed after vocational rehabilitation. The remaining less than 40% of the rehabilitees followed trajectories where either an initial high level of work participation rapidly declined reaching a negligible level one year after vocational rehabilitation or where work participation was at a negligible level already more than one year before vocational rehabilitation and remained at that level. To our knowledge, this is the first study to provide information on latent trajectories of work participation around vocational rehabilitation.

In our data, those who resumed their initial high level of work participation can be considered as the most favorable group with typical return to full duties after sickness absence and the rehabilitation episode. The three less favorable trajectory groups were generally associated with older age, male gender, living in Eastern or Northern Finland, having less than tertiary education, being rehabilitated in the private sector, being rehabilitated due to mental disorders, starting rehabilitation in 2008 instead of in the two later years, as well as being rehabilitated during shorter or longer periods than the typical three-month period.

Those who resumed their initial medium level of work participation can also be considered as a relatively favorable group. This was the group for which partial work disability was most common both before and after vocational rehabilitation. Return to partial duties could have been an alternative to full disability retirement and therefore promoted overall work participation. Indeed, our earlier studies suggest that being on partial work disability prevents later full disability retirement [30,31]. Factors associated with this trajectory in particular were prior employment in the health and social work sector, prior high exposure to kneeling and squatting at work, and being rehabilitated due to neoplasms or injuries.

Among those who had a negligible level of work participation already before vocational rehabilitation, full disability retirement was very common, while the use of partial work disability benefits at any period was virtually non-existent. Factors associated with this trajectory in particular were prior employment in the health and social work sector as well as high prior exposure to job strain. Furthermore, lower education had a particularly strong association with this trajectory.

Those whose initial high level of work participation rapidly declined and, despite vocational rehabilitation, reached a negligible level typically transitioned to full disability retirement or unemployment. This trajectory in particular was associated with prior employment in the manufacturing and trade sectors as well as with being rehabilitated due to nervous diseases.

Previous studies have indicated that previous employment [2–5,7,9] and shorter length of preceding disability [1,5,8] were associated with more favorable employment outcomes after vocational rehabilitation. We nevertheless demonstrated that for a relatively large group of rehabilitees, work participation declined to a negligible level despite a high level of work participation before vocational rehabilitation. Furthermore, another group of rehabilitees even somewhat exceeded their initial medium level of work participation despite commonly having periods of work disability and unemployment before vocational rehabilitation. Previous labor market participation therefore appears to determine the outcomes to a limited extent, and vocational rehabilitation may be successful even among those who are not currently attached to the labor market. Previous findings from Finland indicated that vocational rehabilitation was associated with return to work after temporary disability retirement [32].

In line with our findings, many previous studies also indicated that younger age [1–3,5,7–10] and higher educational level [1–4,6–8,10] were associated with more favorable employment outcomes after vocational rehabilitation. These factors are likely to be associated with better work ability or employment opportunities more generally. In our study, living in Southern or Western Finland was also likely to be associated with better employment opportunities than living in Eastern or Northern Finland, where employment rates were lower [24]. In addition, employment careers were likely to be more secure in the public than in the private sector.

Previous findings from Sweden indicated that work resumption after vocational rehabilitation was higher in the manufacturing industry than in other sectors [18]. Somewhat contradictorily, we found that prior employment in different industrial sectors had heterogeneous influence and prior job exposures had very limited influence on work participation trajectories around vocational rehabilitation. Employment in the manufacturing or trade sectors was associated with the trajectory where an initial high level of work participation rapidly declined to a negligible level. Economic and structural changes during our study period likely led to reduced employment opportunities in the manufacturing sector and changes in the types of jobs available in the trade sector [33]. Those who were exposed to kneeling and squatting and job strain as well as those who were employed in the health and social work sector were the most likely to follow the trajectories where work participation was either at a medium or low level already before vocational rehabilitation. Earlier rehabilitative efforts, combined with employment services that could reduce unemployment after rehabilitation, might therefore be beneficial for these groups of employees. A recent meta analysis on active labor market programs indicated that on the average, programs promoting the accumulation of human capital, such as training and subsidized private sector employment, have better potential to increase employment than other types of programs [34]. More research is nevertheless needed to determine which particular services are most effective for individuals with work disability histories.

Previous studies have found either no effects [1,2,7,9] or mixed effects [3,5,6,8,10] of gender on employment outcomes after vocational rehabilitation. We found that men followed less favorable work participation trajectories. Men could have been employed in segments of the labor market where adapting to new work tasks or occupations was more difficult. Somewhat surprisingly, however, the gender difference persisted even after controlling for education, industrial and employment sectors, as well as different job exposures. Previous studies indicated that men have fewer contacts with health care services [35–37] and lower rates of sickness absence [38–41] than women. The smaller proportion of men in our present study population also reflects their lower participation in vocational rehabilitation. These issues may indicate lower morbidity and better work ability among men, but they may also indicate that men have poorer or delayed access to treatment or that men are less prone to report sick. When men do enroll in vocational rehabilitation, it may be at a later and more severe stage of work disability compared to women.

Differences in employment outcomes after vocational rehabilitation by disease group have also been somewhat unclear. Many studies have focused on particular disease groups [1–3,8,9]. Studies comparing different disease groups found that employment outcomes were more favorable among rehabilitees with physical health problems than among those with mental disorders [6,7,10]. Furthermore, a systematic review focusing on overall workplace interventions directed at workers on sick leave suggested that the interventions may be associated with more favorable labor market outcomes among those with musculoskeletal diseases, but not necessarily among those with mental disorders [42]. In accordance, we found that those with mental disorders followed less favorable work participation trajectories around vocational rehabilitation than those with other diagnoses. We also found that those with musculoskeletal and circulatory diseases followed the average trajectories. Furthermore, those with nervous diseases were more likely than those with other diagnoses to follow the trajectory where an initial high level of work participation rapidly declined to a negligible level. Those with neoplasms and injuries were more likely than average to follow the trajectory where initial medium level of work participation was resumed. Such work resumption can be considered as a successful outcome; the individuals in question may have had a condition severe enough, e.g. cancer or injury, that reduction in work ability due to the condition or its treatment impeded return to normal duties without vocational rehabilitation.

Prevailing economic conditions may influence labor market outcomes after vocational rehabilitation [6,43]. We found that those who started their rehabilitation in 2009 or 2010 were less likely to follow the trajectories where initial work participation was at a medium or a low level than those who started their rehabilitation in 2008, i.e. before the peak of the economic recession. This may have been caused by selection: especially among those with weak work attachment, the recession may have decreased the likelihood of receiving vocational rehabilitation because of increased risks of unemployment and being outside the labor force.

We found that both shorter and longer than the typical three-month duration of vocational rehabilitation were associated with the two trajectories with negligible levels of work participation after rehabilitation. Duration may have been related to the type of vocational rehabilitation received, on which we did not have information. In the Finnish system, work try-outs typically last for a few months, while work counselling typically lasts for six months or more. The duration of training may vary considerably, the content ranging from short courses to training programs lasting for several years [23]. In our data, the vocational rehabilitation episodes could have included successive vocational rehabilitation efforts of different types, making findings relating to the duration of vocational rehabilitation hard to interpret. Furthermore, particularly short durations of vocational rehabilitation may have indicated that the vocational rehabilitation program was interrupted. However, even among those who received rehabilitation for less than three months in our data, 74.3% of the episodes ended because the fixed-term vocational rehabilitation benefit came to an end, thereby not suggesting premature exit from the program. The rest of these short episodes ended because of unspecified reasons, including return to work.

### Methodological considerations

Our study had various strengths. The study population consisted of individuals who were derived from a large nationally representative sample and who had participated in a statutory nationwide program of vocational rehabilitation. The register-based data did not have missing information or loss to follow-up. Moreover, the rich data comprised detailed longitudinal information on employment participation, vocational rehabilitation, other benefit receipt, various sociodemographic factors, industrial sector, and occupation. Using specific occupational codes, the data were further complemented with information on various job exposures based on previously developed job exposure matrices. Furthermore, by using a semiparametric group-based modelling strategy, we provided novel findings on latent trajectory groups of work participation around vocational rehabilitation. Including information on work participation both before and after vocational rehabilitation reduces some of the confounding effect of labor marker history on subsequent labor market outcomes [19].

Our study also had limitations. The pension insurers conducting the vocational rehabilitation do not systematically collect data on the provided services. We therefore did not have information on the specific type of vocational rehabilitation or on characteristics of the provider. Neither did we have information on treatment history nor on potential receipt of medical rehabilitation. In Finland medical rehabilitation is conducted separately from vocational rehabilitation and it is therefore not a part of the vocational rehabilitation process. Labor market outcomes may depend on whether the provided services include e.g. educational, job-related, medical, or other rehabilitation [4,8,18,19,44–46]. Our study focused solely on non-medical, employment-oriented vocational rehabilitation, provided to people with relatively good previous labor market attachment. Results for particular service contents within such rehabilitation may nevertheless largely vary from the presented average ones. Moreover, by excluding vocational rehabilitation episodes lasting more than 22 months, our findings may not apply to prolonged episodes such as those related to successive vocational rehabilitation efforts of different types or to longer-term training programs.

In addition, by using the job exposure matrices, we did not capture variation in the working conditions between individuals holding the same occupational title. The influence of job exposures on the work participation trajectories may therefore have been underestimated.

It was beyond the scope of this study to assess the effectiveness of vocational rehabilitation on work participation. Favorable or unfavorable work participation trajectories do not necessarily mean that vocational rehabilitation was successful or unsuccessful, respectively. Some individuals may still have had good chances of return to work irrespective of whether or not they took part in vocational rehabilitation. Others may have been less responsive to vocational rehabilitation or, for one reason or another, may not have participated in vocational rehabilitation during an optimal time frame. In our data, full disability retirement and unemployment were relatively common already before vocational rehabilitation, suggesting that interventions were not always carried out at early stages of reduced work participation. Further nationwide studies are needed to assess the effectiveness of vocational rehabilitation on work participation outcomes, taking into consideration both individual and work-related factors.

## Conclusions

Work participation trajectories and associated changes in other labor market statuses before and after vocational rehabilitation appear to be diverse. Previous levels of work participation were commonly resumed after vocational rehabilitation, but at the same time work participation could decline to negligible levels despite high initial levels. Less favorable work participation trajectories appear to be generally associated with older age, male gender, living in areas with poorer employment opportunities, lower education, employment history in the private sector, and mental diagnoses.
